# An Introduction to Faculty Diversity, Equity, and Inclusion for Excellence in Nurse Education: Literature Review

**DOI:** 10.2196/49231

**Published:** 2023-12-01

**Authors:** Emily Ganek, Romy Antonnette P Sazon, Lauren Gray, Daisy Sherry

**Affiliations:** 1 College of Nursing and Health Sciences Lewis University Romeoville, IL United States

**Keywords:** nursing education, diversity, equity, inclusion, inclusive, nursing, allied health, medical education, nurse, nurses, health care education, curriculum, DEI, teaching, educator, educators, hiring, recruiting, recruitment, teacher, teachers

## Abstract

**Background:**

The diversity of the world’s population is increasing, along with the health inequities of underrepresented minority populations. To provide high-quality care to all patients, nurses require an understanding of diversity, equity, and inclusion (DEI) as well as how to implement best practices. Nurse educators are the ones to lead the way for DEI education for students.

**Objective:**

This paper aims to describe the findings of a literature review that introduces DEI concepts for excellence in nurse education and their related benefits. Best practices for actions to address DEI in nursing education will be described.

**Methods:**

After institutional review board approval, a literature search yielded 61 articles using 15 distinct keywords in 4 global, peer-reviewed literature databases. Melynk and Fineout-Overholt’s (2023) Levels of Evidence guided the process of selecting 26 peer-reviewed articles and resources.

**Results:**

Common themes for best practices in DEI were identified. These themes included recruiting underrepresented minority nursing faculty, incorporating DEI into an institution’s mission statement, addressing DEI topics in curricula, providing leadership, having a DEI strategic plan, developing education, developing data-based interventions, instilling policy change, partnering in outreach, targeting impact on hiring committees, recognizing DEI work, and providing mentorship.

**Conclusions:**

In summary, this literature review provides several strategies to address DEI for nurse educators. Committing to DEI efforts and improving diversity in the nurse educator workforce are integral steps in improving the quality and inclusivity of nursing education and ultimately improving the health of our communities.

## Introduction

It is well known that the diversity of the world’s population is increasing, along with the health inequities of underrepresented minority populations. To provide high-quality care to all patients, nurses need to understand diversity, equity, and inclusion (DEI) and implement best practices. The American Association of Colleges of Nursing (AACN) [1] declared the need for DEI in nursing academia to better prepare future nurses. Nursing educators are in a prime role to lead education about DEI for the next generation of nurses.

In the United States, the proportion of full-time underrepresented minority nursing educators was 18% in 2019 [2,3]. In the state of Illinois, 20.1% of nurse educators identified as an underrepresented minority, which falls short in contrast to the 31.3% of underrepresented minority nursing students [2]. Efforts to increase the number of underrepresented minority nursing educators must be put into place to better match the needs of the student population. AACN [4] supports expanding the diversity of nurse educators. To strengthen the quality of nursing education in DEI to promote the inclusion of underrepresented students, recruiting more diverse nursing educators is essential. Diverse nurse educators provide a diversity of perspectives and faculty to attract aspiring underrepresented minority students.

The objective of this paper is to provide the results of a literature review that introduces DEI concepts for excellence in nurse education and their related benefits. Evidence-based best practices for actions to address DEI in nursing education will be discussed.

## Methods

### Ethics Approval

The Lewis University Institutional Review Board approved this educational research on March 25, 2022.

### Procedure

A literature review was done to investigate what is known about the attitudes, beliefs, and strategies related to building cultural competence among staff and faculty in a school setting. Primo, ProQuest, Google Scholar, and Mendeley were used to perform the literature review that yielded 61 articles and readings.

The following keywords were searched in variation for this literature review: *cultural competency*, *nurse educators*, *training*, *supporting diverse nursing faculty*, *diversity of nursing faculty*, *nursing faculty diversity*, *inclusivity for nursing faculty*, *holistic review*, *diversity*, *nursing workforce*, *holistic admissions*, *cultural competence*, *nursing*, and *influence on students and diversity.*

Melynk and Fineout-Overholt’s Levels of Evidence [5] were used during the process of finding peer-reviewed articles for this literature review. All articles included were either primary or secondary sources and were written within the last 5 years. The search for DEI concepts was researched first through the AACN. Having a defined set of DEI concepts directed subsequent articles that sought the benefits of diversity in the nurse educator workforce.

## Results

### Overview

The following definitions and suggestions are the result of the literature review. Many resources in nursing education look to the AACN for how to define DEI. These definitions will be provided. “Benefits of diversity in the nursing workforce” was a common theme within each of the articles and resources examined. These benefits will also be provided.

After an examination of the literature, common themes for best practices in DEI were identified. These themes include recruiting underrepresented minority nursing faculty, incorporating DEI into an institution’s mission statement, addressing DEI topics in curriculum, providing leadership, having a DEI strategic plan, developing education, developing data-based interventions, instilling policy change, partnering in outreach, targeting impact on hiring committees, recognizing DEI work, and providing mentorship.

### Defining DEI

The AACN [1] provides important definitions (Textbox 1) for the terms diversity, equity, and inclusion. It is necessary to be knowledgeable of the many aspects that encompass DEI to fully grasp the importance of diversifying the nurse educator workforce. An institution that values and embraces DEI efforts, appreciates opposing opinions, inspires innovative problem-solving strategies, and boosts creativity within the academic institution is more likely to draw diverse applicants [6].

**Definitions of diversity, equity, and inclusion.**

**Diversity**

“A broad range of individual, population, and social characteristics, including but not limited to age; sex; race; ethnicity; sexual orientation; gender identity; family structures; geographic locations; national origin; immigrants and refugees; language; physical, functional, and learning abilities; religious beliefs; and socioeconomic status.”

**Equity**

“The ability to recognize the differences in the resources or knowledge needed to allow individuals to fully participate in society, including access to higher education, with the goal of overcoming obstacles to ensure fairness.”

**Inclusion**

“Environmental and organizational cultures in which faculty, students, staff, and administrators with diverse characteristics thrive.”

Note: Definitions are taken from the American Association of Colleges of Nursing [1].

### Benefits of Diversity in the Nurse Educator Workforce

One key benefit of diversity in the nurse educator workforce is a better representation of the diversity of the student population. An institution with diverse nursing faculty and staff attracts and inspires current and prospective students to identify with their educators. Students associating with faculty whom they identify with promotes a sense of belonging. Alsulami and Sherwood [7] note that underrepresented minority students are motivated by seeing themselves represented by faculty who are successful in their careers. AACN [8] adds that underrepresented minority nursing faculty can inspire students looking to join the nursing profession. Increasing the diversity of underrepresented minority faculty members enriches the cultural climate of a teaching institution. The Center for American Progress emphasizes the importance of recruitment and retention of a diversity of faculty members to combat the imbalance of diversity between students and educators [9].

Guler [10] demonstrates that diverse students need diverse and inclusive teaching approaches. Educators with the best intentions verbalized frustration connecting with a diversity of students. For example, 65% of faculty members reported they did not have enough training to educate culturally and linguistically diverse students properly [10]. Volpe [11] added that underrepresented minority students attending schools with a lack of diversity reported feeling alone and unsupported. To solve this dilemma, Partelow et al [9] stated that recruiting faculty from underrepresented minority groups will help improve culturally responsive teaching. Diversity in language and culture is a positive experience for classroom learning allowing students to learn from both educators and peers. A diversity of nurse educators provides different outlooks, pedagogies, and experiences enriching the education of the students [7]. The ultimate goal would be to recruit a widely diverse faculty to support a diversity of students, increase inclusivity in the organization, and improve the cultural climate.

## Discussion

### Principal Findings

In the search for excellence in the recruitment of diverse nursing faculty, several common strategies were identified. The following practical strategies, created from a thorough literature review, can be applied to any school of nursing for the creation of a more culturally inclusive environment that supports a diversity of nurse educators.

#### Mission Statement

A mission statement conveys the purpose and intentions of the group and sets the tone for the group culture. A clear mission statement for nursing regarding DEI is critical for understanding the culture, holding leaders accountable, and directing the learning environment [4]. Therefore, the first step in creating a more diverse nursing faculty is a mission statement driven by DEI principles. By doing so, a mission statement dedicated to providing culturally competent education is indisputable. Moreover, Alsulami and Sherwood [7] found that faculty development offerings led to greater job satisfaction for underrepresented minority faculty when the mission and culture of the university were highlighted. The mission statement is the nucleus of an institution, guiding all actions.

A mission statement that describes values in DEI also helps support and sustain faculty work. Retention rates for underrepresented minority faculty were increased in institutions with mission statements embedding DEI values [7]. However, it is important to discern that simply rewording a mission statement to add popular DEI catchphrases will not be effective. Having core DEI values requires measurable initiatives for call-to-action stances on social justice to mean something [12]. This means that a mission statement driven by DEI principles is a first step, which then needs to be the anchor for a school’s set of priorities, resources, and decisions, and in turn, be evident in the campus climate [13]. For example, the University of Pennsylvania School of Nursing exhibits its commitment to diversity through its mission statement, its Office of Diversity and Inclusivity’s strategic goals to prioritize diversity in the social and cultural life of the campus, and its attentiveness to adding cultural competence into the curriculum [14]. The mission statement of a school is a vital message to the community.

#### Curriculum

The incorporation of DEI concepts within the curriculum in nursing programs is a key component in attracting diverse faculty members. For example, a community health course can concentrate its clinical portion of the course on providing care to historically underserved populations, while continuing to practice their fundamental skills [13]. In another instance, in a nursing research course, a class assignment could incorporate the identification of DEI topics, retrieve related research articles, create in-class discussions, and participate in reflection activities about their given topic [15]. It was found through student course evaluations that incorporating DEI helped create a more inclusive learning environment and supported their personal growth. A more diverse group of nursing faculty and students cannot be accomplished without intentionally including DEI concepts in the curriculum and learning environment [16].

Syllabi within the nursing curriculum communicates class expectations. Among these expectations, the creation of an inclusive learning space is fundamental. The learning space includes the physical classroom, internet-based classroom, and experiential clinical setting. For example, Lewis University promotes a sanctified zone where learning spaces encourage diversity and oppose bias and prejudice [17]. Communicating a persistent and consistent inclusive message to the students and community in course syllabi is a practical way to support faculty diversity and recruit potentially underrepresented minority faculty. Prospective underrepresented minority faculty will find value in the addition of DEI concepts in the curriculum on which they base their work.

Loyola Marymount University’s School of Education stands as an example to other universities for how they prepare their educators to serve a diverse student body. For example, a significant portion (40%) of their teaching competencies are specifically related to diversity [9]. Examples of diversity-specific teaching competencies incorporate advocacy for social justice and inclusion, respect for all individuals, and leadership that is moral and caring [9]. Having the next generation of educators meet these competencies demonstrates a commitment to diversity and ensures educators have the ability to teach in an inclusive way. A diversity of prospective faculty will see that the school of nursing values DEI, as stated in their mission, when the curriculum provides learning opportunities regarding DEI efforts.

#### Academic Leadership

Leaders in academia uphold the mission statement of the learning institution. In doing so, the cultural expectations of faculty are outlined. However, it is known that unconscious bias is a characteristic of being human. Everyone has their own biases. Unfortunately, biases from academic leaders or faculty members can unintentionally hinder the ability to recruit diverse nursing faculty members [1]. It is recommended that all leadership and faculty members increase their self-awareness of bias and reflect on how that bias could impact a nursing school’s endeavor to improve DEI efforts. More importantly, it is recommended to reflect on how bias can be overcome to promote DEI efforts.

Strategies to overcome bias include self-assessments on awareness and identification of individual biases. Subsequently, education and support to empower faculty with knowledge for personal and professional development is a responsibility of leadership. Resources, continuing education, and readings on inclusivity and bias are helpful for all faculty members to learn and grow. Byrd et al [18] highlight the importance of self-study on the topic of inclusivity and actively exposing oneself to diverse viewpoints. Inclusive leadership includes a diversity of perspectives for making decisions. Inclusive leaders promote diverse thinking while ensuring a respectful environment for discernment.

Along with biases, lack of support is another barrier in leadership for the recruitment and retention of diverse nursing faculty members. Iheduru-Anderson et al [19] found that many Black nurse leaders felt a lack of support from the institution’s leadership. Actions such as the absence of academic leadership at important DEI events conveyed the message that these events are not significant [19]. AACN [4] highlights the importance of leadership participating in DEI activities and evidence-based training to ensure that the school can fulfill its commitment to advancing the quality of nursing education regarding DEI. An academic leadership team that is visible and campaigns for DEI initiatives will assist in recruiting diverse faculty members.

There are many other ways a leadership team can demonstrate a positive cultural climate for the promotion of diversity. Embracing differences, ensuring proper work-life balance, valuing a diversity of educators’ voices through inclusion in decision-making procedures, and rewarding faculty members for DEI-related work are a few examples [7]. Leaders at the highest level of the institution must consistently convey the importance of diversity and inclusion to support underrepresented minority faculty to feel “that their presence on campus is part of the collective group, not a representation of the invisible few” [7,10]. In other words, the value of a diversity of underrepresented minority faculty members should be evident in all facets of the institution and continually reinforced by leadership.

#### DEI Strategic Plan

A DEI strategic plan is a document that communicates the strategies and goals regarding DEI efforts of the learning institution and provides ways to become a more inclusive and equitable environment. A DEI strategic action plan will identify stakeholders, set goals, create objectives, and develop outcomes and metrics that all align with the mission of the institution [4]. One of the first actions to include in a DEI strategic plan is the creation of a DEI committee to facilitate efforts [11]. The addition of a DEI leader can also assist with coordinating efforts between departments and the university, serving as a resource for mentorship and faculty advice, promoting a welcoming culture for a diversity of educators, and leading the nursing school’s DEI committee [4]. The DEI strategic plan and DEI committee are a powerful duo. Prospective and current nursing faculty will appreciate the guidance of a clear plan and feel supported by the DEI committee members.

The University of Pennsylvania School of Nursing proves to be a model for excellence in diversity and inclusion as demonstrated by the key performance indicator of faculty diversity. As of 2018, the University of Pennsylvania School of Nursing was one of 3 schools to have an Office of Equity and Inclusion [14]. This office focuses on the promotion of classroom strategies to ensure culturally sensitive and inclusive teaching-learning strategies and provides support to underrepresented minority faculty and students. As a result, the University of Pennsylvania School of Nursing has achieved a faculty diversity rate of 24.5% underrepresented minority faculty members [14]. This percentage is notably greater than the 2019 national average of 18% full-time diverse faculty members [3]. The University of Pennsylvania School of Nursing introduced a position called the Diversity Search Advisor to create hiring goals and strategies for promoting diversity. Some of these strategies include organizing implicit bias workshops for faculty search committees, improving university policies that relate to diversity and inclusion, and scheduling discussions about how to increase the pool of diverse applicants [14]. These strategies display the school of nursing’s commitment to diversity, which has been successful in increasing faculty diversity.

#### Faculty Education

Although a DEI strategic plan is key for an organization dedicated to the promises of providing fair opportunities for all students, it is important to take it a step further and ensure that all faculty and staff are fully knowledgeable to implement these actions. AACN [4] prepared a DEI Tool Kit to guide and assist schools of nursing with implementing DEI strategies, expanding the capacity of nursing faculty to grow professionally and personally. Evidence-based resources and exemplar models are provided to give faculty the tools necessary to build an inclusive teaching and learning environment [4]. For example, the DEI Tool Kit contains an area called “Access and Success” that recommends developing a recruitment plan to expand the diversity pool for the next generation of nurses, creating a pipeline program for underrepresented minority high school students, and establishing articulation agreements from associate degree and baccalaureate degree programs through graduate studies to promote educational advancement. There are 3 additional areas in the DEI Tool Kit—Institutional Viability and Capacity, Climate and Intergroup Relations, and Education and Scholarship—providing a variety of strategies to advance DEI work. To support faculty education related to the DEI Tool Kit, AACN offers a series of free Inclusive Excellence webinars.

Research shows that many faculty members have a low sense of self-efficacy related to teaching DEI concepts [20]. One way to implement faculty education is to start with new faculty orientation that sets expectations for the faculty role in optimizing the learning space as inclusive and safe. Subsequent educational offerings can be expected on a regular basis as one way to meet the requirements for promotion and tenure. These offerings for DEI education should include current faculty members. Institutions that used educational interventions for DEI training saw more student success, increased faculty involvement, and positive faculty feedback [7]. O’Connor et al [20] conducted an incentivized 3-day DEI institute to give faculty the knowledge and skills to embrace DEI concepts and create culturally competent teaching practices. The offering of the DEI institute found statistically significant increases in faculty and staff self-efficacy in teaching DEI-related topics [20]. Many of the participants felt that the training would improve their teaching and that DEI-based learning is valuable to consider in yearly faculty competencies.

Iheduru-Anderson et al [19] suggested that cultural competency education should cover topics such as racism, microaggressions, diversity, and inclusion. It is important that these sensitive topics are covered in separate interactive and introspective educational modules to promote self-awareness, empathy, and compassion for others. An institution that offers continuing education to faculty on DEI concepts is attractive to prospective and current underrepresented minority faculty to promote inclusion and enhance knowledge, thereby assisting in the recruitment and retention of faculty.

#### Data

Another key step for excellence in the recruitment of underrepresented minority nursing faculty is the use of data. Data are well known to drive institutional decision-making. Collecting data regarding faculty and student recruitment practices, acceptance rates, retention rates, and progression to measure and compare performance among groups will be informative for making decisions [11]. Metrics keep track of performance and assessments of the current climate. Some examples of metrics and data are the Center for Urban Education’s Racial Equity Tools, climate surveys, and governance committees’ feedback.

The University of Southern California’s Center for Urban Education developed Racial Equity Tools, broken down into 4 phases, with the goal of improving racial justice in education [21]. The University of Southern California uses these tools to identify gaps in practices, policies, or individual beliefs to promote equity in the educational outcomes and opportunities for underrepresented minority students [21]. These tools are user-friendly and able to be used without a facilitator’s assistance. Data results inform the institution’s ability to learn about its areas of success and growth.

Another helpful metric for data on performance is an institutional climate survey. Climate surveys measure the perceptions of an institution’s culture and provide a voice for participants to offer feedback as to what the institution is doing well, where there is room for improvement, and how the institution supports DEI efforts. To change the climate of academic nursing to value inclusion and diversity, the experiences of those impacted by these matters should be assessed through climate surveys. Climate surveys are deployed on a routine basis to provide a snapshot of the current climate and gather comparative data over time. Positive results from cultural climate surveys lead to greater retention of underrepresented minority faculty [22]. Similar to the Racial Equity Tools, a climate survey allows institutions to identify both areas of success and growth.

Viewpoints from current students, faculty, and staff members provide important details on the institutional culture and tips for addressing areas for growth. Byrd [13] found that nursing education programs successful at increasing DEI efforts obtain students’ input often through governance committees and focus groups. This information is used to make changes, whether changes to outdated policies or enhancements in the classroom to promote effective teaching strategies. Data and feedback help to inform institutions about the need to create a diverse and inclusive academic environment.

#### Policy Change

Institutional, departmental, and programmatic policies need to reflect the commitment to DEI and support the DEI strategic plan. Policies and procedures regarding recruitment, acceptance, and progression of both faculty and students are of particular importance [11]. Taking action to address outdated policies and procedures speaks volumes to the perceived value and commitment to DEI work. According to AACN [4], there should be a continual review of current policies to remove barriers that prevent the advancement of DEI efforts. Examination of policy language is recommended for the reduction and elimination of any systemic bias or prejudice.

The gap in diversity between the educator workforce and the student population is wide. Policy changes regarding the improved recruitment and retention of underrepresented minority faculty members could help lessen this gap [7,9,11]. Institutional policies and key performance indicators need to involve inclusion and equitable work environments. For example, learning institutions could incorporate inclusive procedures to hire based on merit while also mitigating bias in search committees. Reducing bias includes addressing any prejudgments related to age, race, gender, or other characteristics that are not related to performance.

#### Outreach

The establishment of strategic partnerships throughout the community is a key step in increasing the pipeline of prospective underrepresented minority faculty members [11]. For example, the University of Pennsylvania School of Nursing uses the Diversity Search Advisor to represent the school of nursing at outreach events, internal and external meetings, and consultations with a diversity of community and educational leaders to attract a diversity of student and faculty applicants [14]. It is helpful for a leader who is knowledgeable and comfortable with DEI concepts to promote community partnerships. Along the same lines, Teach for America strategies for recruitment can be helpful [9]. The members of Teach for America have a role in expanding awareness of goals for diversity and advocacy of inclusion for prospective applicants. For example, campuses with a high percentage of a diverse student population, accredited historically black colleges and universities, and Hispanic-serving institutions are ideal places to form relationships with future students and educators [9].

The most effective nursing programs integrate outreach with recruitment. For example, in the United States, every public school district’s data on student diversity are made available to the public. Nursing leaders and DEI experts can select to partner with schools that serve a diverse student body. Increasing the number of underrepresented minority nursing students ultimately increases the number of potential future underrepresented minority nurse educators.

#### Hiring Committee

Displaying diversity in the search committee and using a holistic hiring process are two key steps to recruiting more diverse nurse educators. AACN recommends a guide provided by Columbia University as an exemplar for best practices in faculty search and hiring [4,23]. Columbia University [23] recommends that the recruitment and hiring of diverse candidates should be done in a space where the hiring committee is also diverse. In line with this, a web-based survey found that nurse educator applicants from underrepresented minority groups would prefer to see a more diverse hiring committee that is a better representation of themselves during the interview process to foster a sense of belonging [24].

Using a holistic hiring approach includes an examination of the applicant as a whole person. A holistic hiring approach considers more than a narrow view of the person’s professional skill set. Rather, the holistic hiring approach will examine the applicant’s values, how they interact with others, and their potential longevity with the institution. Incorporating a holistic hiring approach can prove beneficial for the overall culture of the workplace.

#### Recognition of Work

The role of faculty members includes not only teaching and scholarship but also university service, such as participating in DEI committees or mentoring programs. Service contributions, when speaking of promotion and tenure, are given less weight compared to research, teaching, or clinical practice [13]. DeWitty and Murray [22] report underrepresented minority faculty often serve on DEI committees and participate in DEI events, but this work is not as highly valued as research or scholarship. As a result, underrepresented minority faculty often feel unsupported and have less time for promotion, tenure, and professional development [7]. DEI work is important to driving the learning institution’s mission and needs to be counted as valuable to career advancement. Giving credit to DEI work will support faculty retention, promotion, and tenure. It is recommended to give greater weight to activities that promote and influence diversity in an institution in the promotion and tenure process [13].

Iheduru-Anderson et al [19] report on the negative experiences of Black women in academic nursing and the increased responsibilities assumed of them. For example, it was reported that colleagues often expected the Black nurse educator to be the leader of diversity and inclusion committees or the planner of cultural competency training, without providing proper resources. From the mission statement to the promotion and tenure process, a just and inclusive culture provides support and welcomes all to the role and shared responsibilities of being a faculty member within the nursing community.

Indiana University-Purdue University Indianapolis (IUPUI) is one of the first institutions to begin compensating employees, through tenure and promotion, for DEI-related work [25]. IUPUI’s policy gives credit to accomplished work that strengthens the institution’s efforts toward being inclusive and rewards those contributing to DEI. For example, an African American assistant professor at IUPUI described how she was asked to participate in many DEI-related roles, and because of the policy change, her research on DEI excellence in service counts for promotion [25]. It is well known that promotion and career advancement are key factors in attracting faculty. In the recruitment of diverse faculty members, the importance of recognition for DEI-related work should be highlighted, especially in terms of promotion and tenure.

#### Mentorship

Research shows that faculty members value the support of mentorship. There are data supporting the number of underrepresented minority faculty lost to tenure rejection or attrition [25]. Byrd [13] found that a significant factor in the retention of underrepresented minority faculty is offering a high-quality mentoring program. Mentorship has been shown to increase a sense of belonging, confidence, and support while fostering an environment of equity where equal pay and promotion occur fairly [26,27]. Mentorship would provide professional and personal support, in turn preventing attrition.

One exemplary organization for mentorship is Penn State, whose nursing program provides mentorship programs, faculty development opportunities, training, and workshops [14]. Another impactful factor for the retention and recruitment of faculty includes support from colleagues and leaders [7]. Forms of support include mentorship, quality new employee orientation, and continuing faculty development. Having a multitude of options for faculty to connect and learn with and from each other is invaluable.

### Conclusions

Diversifying the nurse educator workforce is an essential strategy for creating a more equitable environment. The increasing diversity of nursing students and the next generation of nurse educators requires nursing education to evolve. Adopting best practices for the recruitment and retention of underrepresented minority faculty members will expand the pipeline of nurse educators. Committing to DEI efforts and improving diversity in the nurse educator workforce are integral steps in improving the quality and inclusivity of nursing education, ultimately improving the health of our communities.

## Abbreviations

AACN: American Association of Colleges of Nursing
DEI: diversity, equity, inclusion
IUPUI: Indiana University-Purdue University Indianapolis
